# Validity, reliability and clinical utility of ASSIST-Y in assessing risk of substance-related harm and dependence in Spanish male adolescents

**DOI:** 10.1186/s13034-024-00845-6

**Published:** 2025-01-13

**Authors:** Núria Ibáñez-Martínez, Matthew William Richard Stevens, Núria Civit-Bel, Noemí Moreno-Ferrer, Sandra Lopez-Ferré, Ana Olivares-Casado, Juame Claramunt-Mendoza, Chris Holmwood, Robert Ali

**Affiliations:** 1 Departament de Justicia, Drets I Memoria. Direcció General d’Execució Penal a la Comunitat i de Justícia Juvenil, Barcelona, Spain; 2 School of Biomedicine (Pharmacology), The University of Adelaide, Adelaide, Australia; 3 Parc Sanitari Sant Joan de Déu, Barcelona, Spain; 4 Helen Mayo South Building, 1 Frome Road, Adelaide, South Australia 5005 Australia

**Keywords:** Adolescent, Screening, ASSIST-Y, Polysubstance use, Dependence

## Abstract

**Background:**

Substance use among adolescents is strongly associated with adverse physical, mental health, and social outcomes. Prevention and early intervention can reduce the likelihood of future problems, but requires valid and reliable screening tools capable of assessing risk across a range of substances. This study assessed the validity, reliability, and clinical utility of the Alcohol, Smoking and Substance Involvement Screening Test (ASSIST-Y) for adolescents aged 15–17 years.

**Methods:**

A sample of adolescent males (*N* = 101), aged 15–17 years, held in a juvenile detention facility on substance-related offences in Barcelona, Spain were eligible. Participants were administered a battery of standardized substance-use screening tools by a clinical psychologist, and underwent a diagnostic interview assessing DSM-IV-TR substance abuse and dependence by an addiction medicine specialist. Scores on the various assessments were compared to establish validity (concurrent with interview, convergent with other measures), reliability, and clinical utility of ASSIST-Y.

**Results:**

Majority of participants (*n* = 77) completed assessments. While tobacco was not assessed as part of the interview, concurrent validity in detecting substance abuse was established for all remaining substances. Concurrent validity for detecting dependence was established for alcohol, cannabis, cocaine, stimulants and sedatives. Fewer numbers in higher-risk groups for inhalants, opioids and hallucinogen use limited confirmation of validity for those substances. ASSIST-Y also demonstrated good convergent validity with the other screening tools for all substances, except hallucinogens. Reliability for each subscale was established, except for tobacco (too few items), sedatives, and hallucinogens. Finally, clinical utility indices were significant for most substances (except sedatives and opioids); whilst clinical utility indices were significant for ruling out cases of non-dependence (all substances).

**Conclusions:**

As a screening tool, the purpose of ASSIST-Y is designed to help identify adolescents who may be at-risk of substance-related harm. While the instrument was found to be valid and reliable in identifying risky use across a variety of substances, further research is needed to validate the instrument in other population groups, and for other substances. Future research should investigate the effect of the linked brief intervention to reduce risk of harm, especially for non-specialist clinicians.

**Supplementary Information:**

The online version contains supplementary material available at 10.1186/s13034-024-00845-6.

substance use disorder, alcohol, tobacco, illicit substance use, cannabis.

## Introduction

Alcohol, tobacco and other drug use during adolescence is strongly associated with the development of disability, premature mortality, and other adverse outcomes [1]. Excessive substance use during adolescence, beyond impairing the developing brain [2], is linked to a range of social issues [3], heightened risks of a number of physical and psychological harms [4–6], and is both a driver and a consequence of delinquent behavior [7]. Evidence also suggests the period of adolescence itself is a critical window for the development of substance dependence [5, 8, 9]. Identifying and responding to adolescent substance use through early intervention is critical to minimizing the risk of future harm.

One of the main challenges in addressing adolescent substance use is the increased experimentation, leading to higher rates of poly-, rather than single-substance use [10] among this age group. Alcohol, tobacco, and cannabis are the most common substances initiated during adolescence, with estimates suggesting over half of adults who use alcohol or other drugs initiate use before age 20 [11]. Evidence from global surveys consistently highlights adolescence as the peak period for substance use initiation, with overall quantity and frequency of use typically increasing until very early adulthood [12, 13]. From a public health perspective, it is important to recognize and understand consumption patterns among adolescents in order to inform population and targeted prevention and early intervention approaches [14].

Early initiation and concurrent use of multiple substances are strong predictors for later substance-related problems [15–18], emphasizing the need for early interventions to mitigate the risk of severity and persistence [19]. However, despite the prevalence of substance use among adolescents, harmful use can often go underdiagnosed in non-substance-related treatment settings [20, 21]. To improve identification and responses to substance use, it is important that valid, and reliable screening tools are available, which can be easily administered across a variety of healthcare settings [22].

While several instruments are available and used widely across different settings, most are designed to assess a single substance, and/or focus narrowly on frequency or quantity of use as markers for dependence. This limits their utility for use in adolescents; given the higher prevalence of hazardous but not dependent polysubstance use. One instrument, however, is designed to assess risk of harm from use of all psychoactive substances concurrently. The Alcohol, Smoking, and Substance Involvement Screening Test (ASSIST) was designed for use with adults in primary health care settings to detect hazardous and harmful substance use that may otherwise go unnoticed [23]. It is typically quicker to administer than existing tests, and has been validated for use in a wide variety of cultures [24]. The ASSIST allows clinicians to assign a risk score for each substance used, facilitating brief interventions based on the recorded risk categories, thereby addressing substance use in a timely and culturally sensitive manner.

ASSIST-Y is a derivative of the ASSIST, designed specifically for adolescents [25]. There are two versions of the instrument, designed for use among children (aged 10–14 years), and adolescents (aged 15–17 years). However, the psychometric properties, including the validity and reliability of ASSIST-Y have so far only been investigated among a sample of Swedish adolescents, and only for alcohol [26]. The psychometric properties of the instrument for the remaining substances, and in other languages have yet to be established. Optimal cut-points segregating low-risk from moderate-risk, and moderate-risk from high-risk are also unknown.

In light of the need for valid and reliable screening instruments for detecting risky substance use in adolescence [22], the aim of this study was to assess these aspects among a sample of adolescents aged 15–17 years. This study had several specific aims. First, it aimed to assess the reliability and validity of ASSIST-Y in identifying cases of clinical substance use disorder or dependence compared with a gold-standard diagnostic interview. Second, it aimed to assess the performance of ASSIST-Y against other commonly used screening tools assessing risk of harm and dependence. Finally, given its purpose as a screening tool, it also aimed to assess the instrument’s clinical utility in identifying cases where an adolescent may be at risk of harm, and to rule out cases of non-dependence. The study was conducted as part of a broader program aimed at improving substance-related health and wellbeing outcomes for minors in detention.

## Methods

### Participants and sampling

Participants for this study included adolescent males housed in a juvenile justice education centre for minors and young men (aged 14–18 years). Eligibility criteria included any males aged 15–17 years who had been detained on a drug-related offence, or under the influence of a psychoactive substance at the time of their arrest; or who were in possession of an illicit substance (*n* = 187). Participants were then excluded if they met any of the following criteria: had already been detained for a period of 3-months or more (*n* = 27); were to be detained for a period of less than 15-days (*n* = 54); were younger than 15 years (*n* = 3), or older than 17 years (*n* = 17); were experiencing acute psychiatric symptoms at the time of recruitment (*n* = 2); had cognitive impairment or intellectual disability (*n* = 2), or had language-barrier issues preventing them taking part (*n* = 3). In five cases, the parents of the minor did not consent to their child’s participation. This left a total of 101 participants enrolled into the study (noting that some participants met multiple exclusion criteria).

## Study design

### Recruitment

Participants were recruited from the Can Llupià Educational Center in Barcelona. Participants were approached directly by a member of the research team and informed of the study’s aims and objectives. Participants were given an opportunity to consent, or to decline participation without penalty. Recruitment occurred between December 1, 2021, and September 30, 2022.

### Procedure

Once informed consent had been established, each participant was administered a series of questionnaires by a registered clinical psychologist. Questionnaire data related to demographics, socioeconomic status, family history, immigration history, and psychological symptoms. The questionnaire also included a series of instruments related to their alcohol and other drug use, including the ASSIST-Y, and four other standardized measures of substance use/dependence. To provide an external reference for assessing validity, all participants were also required to undergo a separate diagnostic interview with an independent addiction medicine specialist (who was blinded to the outcomes of the previous questionnaire). Since baseline and interview data were collected at different times during the study, and some participants moved locations during their stay, not all participants were able to complete both assessments.

## Study measures

### Index screening tools

#### ASSIST-Y (15–17 years)

ASSIST-Y is a screening tool for adolescents, that captures risk of harm across nine commonly used substances [25]. The instrument contains 7-items which relate to a variety of substance-related harm indicators. Question 1 is an initial screener that identifies whether the individual has ever used a given substance. Questions 2–6 capture frequency of use and harm, and Question 7 finally assesses additional risks from injecting drug use. Scores from Q2-6 are summed to provide a total substance specific involvement (SSI) score for each substance, which are then grouped into low, moderate and high-risk, depending on the substance. The ASSIST-Y was developed for use in adolescent populations, and the English version has been validated in a sample of Swedish adolescents previously [26]. For the purposes of this study, the instrument was translated into Spanish using the recommended protocol developed by the World Health Organization [27].

#### Cannabis abuse screening test (CAST)

The Cannabis Abuse Screening Test (CAST) [28], is a six-item self-administered screening questionnaire measuring the frequency of several markers of cannabis use disorder. The instrument is scored using a five-point Likert scale, ranging from 0 (Never) to 4 (Very often). Total scores are stratified into low-risk (≤ 2), moderate-risk (3–6), or high-risk (≥ 7). The Spanish version has been previously validated and is used widely in population surveys [29, 30].

#### Severity of dependence scale (SDS)

The Severity of Dependence Scale (SDS) [31] is a five-item self-administered questionnaire focused on the frequency of experiencing psychological aspects related to risky use in the past year. Each item is scored using a four-point Likert scale, ranging from 0 (Never/almost never) to 3 (always). Total scores are summed, with recommended cut-offs for dependence varying between 3 for alcohol [32], to 7 for benzodiazepines [33] out of a possible 15. A cut-off score of 4-or-greater was chosen for this study to indicate possible dependence for any substance. Previous studies involving Spanish populations have found SDS to demonstrate adequate reliability for all substances [34].

#### Car, relax, alone, forget, family or friends troubles (CRAFFT)

The CRAFFT is a screening tool for assessing alcohol and other drug use in adolescents [35]. CRAFFT consists of six items enquiring about substance use in six situations where it is more common in adolescents. CRAFFT items are scored on a dichotomous ‘yes/no’ format, with the total score ranging is 0–6. Scores of 0, 1, and 2-or-more indicate low, moderate and high-risk of dependence respectively. A Spanish version of the CRAFFT has been validated recently [36]. A revised version of the CRAFFT has been recently developed, however, the original version was used in this study.

#### Fagerstrӧm Test for Nicotine Dependence (FTND)

The Fagerstrӧm Test for Nicotine Dependence (FTND) is a standard instrument for assessing the intensity of physical addiction to nicotine [37]. It contains six items that evaluate the quantity of cigarette consumption, the compulsion to use, and dependence. In scoring the FTND, yes/no items are scored from 0 to 1 and multiple-choice items are scored from 0 to 3. The items are summed to yield a total score of 0–10. Higher scores indicate greater severity of nicotine dependence. Importantly for our study, since the gold-standard reference tool does not assess nicotine dependence, the FTND was used as the reference instead.

### Clinical reference (gold-standard)

#### MINI-Plus diagnostic interview for DSM-IV-TR substance use disorders

The MINI International Neuropsychiatric Interview (MINI) is a structured diagnostic interview designed to assess DSM-IV-TR and ICD-10 diagnoses. MINI-Plus, an extended version of MINI [38], was used in this study to provide a clinical reference point for the ASSIST-Y and other index screening measures. The MINI-Plus has reliable psychometric properties, and is widely used to support diagnostics in psychiatry as the gold-standard for reference for clinical diagnoses [38]. The Spanish-language version of MINI-Plus [39] was used in this study to provide diagnoses for current and/or lifetime abuse or dependence to a range of different drugs, including alcohol, cannabis, cocaine, stimulants, sedatives, inhalants, hallucinogens or opioids. The interview was conducted by an independent trained addiction medicine specialist.

## Statistical analyses

A table summarising the entire statistical approach, as well as a comprehensive narrative account of the statistical approach taken to assess validity, reliability and clinical utility can be found in supplementary materials. This information outlines the tests and parameters used, the thresholds for validity, reliability and clinical utility; as well as the minimum sample size required (based on an a priori power of 1-*β* = 0.90 and a type-1 error rate of *α* < 0.05) to detect an effect at the ‘acceptable’ level (typically corresponding to a small effect). These details are also summarized in the sections that follow. All analyses were conducted in R-studio (version 4.3.2) [40].

### Primary outcomes: validity and reliability

#### Internal consistency reliability

Internal consistency reliability was assessed for each ASSIST-Y subscale using Cronbach’s alpha. Coefficients were bootstrapped (*n* = 1000 samples) to account for low number of scale items (*n* ≤ 5). Items were also standardized to account for the variation in score weightings (see supplementary materials for more information about the need for bootstrapping and standardization). Bootstrapped alpha coefficients ≥ 0.70 were deemed acceptable indicators of reliability.

#### Cross-method agreement

Cross-method agreement refers to the degree of consistency or agreement between different methods or instruments measuring the same construct. For this study, agreement was assessed between ASSIST-Y risk determinations, and MINI-Plus diagnostic classifications, by cross-tabulating and comparing proportions within each outcome. For DSM-IV substance abuse, proportions of those at moderate-risk (versus low-risk) were compared against proportions of those diagnosed with lifetime or current substance abuse (versus no abuse; cases of high-risk use and dependence were excluded from this analysis). Similarly, cross-method agreement for dependence was assessed by comparing proportions of those rated as high-risk (versus low/moderate) to those with lifetime or current dependence. Cohen’s Kappa statistics, with 95% confidence intervals and tests of significance were also reported (see supplementary material for additional information). Strength of cross-method agreement was determined by previous studies [41]. Significant effect sizes of κ > 0.40 indicated acceptable cross-method agreement.

#### Concurrent validity (against gold-standard)

Concurrent validity assesses the extent to which scores on one instrument align with outcomes from a gold-standard reference. In this study, mean ASSIST-Y Substance Specific Involvement (SSI) scores were compared between outcomes from the diagnostic interview. Two-tailed paired-samples t-tests (with 95% confidence intervals) compared score distributions, with Hedge’s *g* coefficients reported as the measure of effect size. Cohen’s standards for determining small, medium and large effect [42, 43] were used. Significant effect sizes of *g* > 0.20 indicated acceptable concurrent validity.

#### Convergent validity (with standardized measures)

Convergent validity assesses the extent to which two (or more) instruments, which are designed to measure the same construct, actually do. This study assessed convergent validity through correlations between ASSIST-Y SSI scores and scores on the other standardized measures (SDS, CRAFFT, and CAST). ASSIST-Y SSI scores were compared to total scores on the other standardized screening tools using a Pearson’s *R* correlation matrix. The strength and direction of correlations providing an indication (or absence) of validity. Since CRAFFT does not assess risk for tobacco, this study focused only on illicit substances and alcohol for this comparison. Cohen’s standards were used to determine weak (*r =* .20-0.49), moderate (*r* = .50-0.79), and strong (*r* > .80) coefficients [44]. Significant correlations of *r* > .20 indicated acceptable convergent validity.

### Secondary outcomes: diagnostic accuracy and performance

#### Diagnostic accuracy

Given ASSIST-Y is designed to identify individuals who may be at risk of harm (for the purposes of a brief intervention), and dependence (for the purposes of referral), a range of diagnostic accuracy indices were assessed for the instrument at various cut-points. The diagnostic interview was used to establish the proportion of true positives (TP), false positives (FP), true negatives (TN) and false negatives (FN), across all substances, except tobacco (where FTND was used instead).

Based on the proportion of true and false positives/negatives, the following diagnostic accuracy metrics were calculated for each substance: sensitivity (TP/FP + TN); specificity (TN/FP + TN); positive predictive value (PPV; TP/FP + TP); negative predictive value (NPV; TN/TN + FN); area under the Receiver Operating Characteristics (ROC) curve; likelihood Ratios for positive (LR+) and negative (LR-) tests; Clinical Utility Index for positive (CUI+) and negative (CUI-). Cut-off scores were established at the point where Youden’s *J* index was maximized. Cut-off scores for low, moderate and high-risk were determined using ROC curves, with areas under the curve (AUC) reported for each comparison. A conservative AUC of ≥ 0.70 indicated at-least acceptable accuracy. A detailed summary of the diagnostic accuracy measures is outlined in the supplementary materials.

#### Diagnostic performance

The final step of the analysis involved comparing the performance of ASSIST-Y with other scales (SDS, CRAFFT, CRAFFT, and FTND) for identifying cases of dependence, and screening out cases of non-dependence. Diagnostic accuracy measures reported above, were also calculated for all scales. Performance was judged based on scales with the highest CUI- and CUI + scores (see supplementary materials for a detailed summary).

## Results

### Sample characteristics

A total of 101 adolescents, aged 15–17 were enrolled into the study and completed the baseline assessments between February, 2022 and December, 2022. Of those completed the questionnaires, twenty-three participants subsequently changed detention centres during the study and were unable to complete the diagnostic interview, while one participant elected to withdraw their participation. This left a total of 77 participants who completed both the assessment battery and the diagnostic interview.

Table 1 summarizes the descriptive characteristics of the total sample. All participants were male, and on average, participants were 16.3 years old, and predominantly originating from either Spain (37.6%), or Northern Africa (41.6%), with nearly half (44.0%) arriving intoxicated at the time of arrest. Nearly one-third (35%) reported having a parent with a substance use disorder, which was predominantly the father (77.1%), and either alcohol (34.3%) or cannabis (31.4%) related. The majority of participants (73%) had previously been diagnosed with an addictive disorder, while many also had either mothers or fathers with an addictive disorder (7%, and 13% respectively). Some (13.7%) reported having a friends or other acquaintances with addiction, while nearly two-thirds (62.7%) had immigrated recently, aged on average 11.5 years old. Nearly half (45.3%) had immigrated alone, of which nearly half (45.3%) reported initiating substance use during their immigration, of which cannabis (43.3%) was the primary drug.

**Table 1:** Descriptive characteristics of sample

	N (%)
Mean Age (SD)	16.3 (0.8)
Region	
Spain	38 (37.6)
Northern Africa	42 (41.6)
South America	15 (14.9)
Eastern Europe	2 (2.0)
Sub-Saharan Africa	1 (1.0)
Other	3 (3.0)
Intoxicated at moment of arrest	44 (44.0)
Offending under influence	62 (60.8)
Parent with SUD	35 (35.0)
Diagnosed with addictive disorder	73 (36.3)
Has network of addiction	14 (13.7)
SDS	
Number classed as *dependent*	73 (72.3)
Number classed as *non-dependent*	28 (27.7)
CRAFFT	
Number classed as *at-risk*	88 (87.1)
Number classed as *not at-risk*	13 (12.9)
CAST	
Number classed as *high risk*	77 (76.2)
Number classed as *moderate risk*	6 (5.9)
Number classed as *low risk*	2 (2.0)
Mean (SD) substances used in lifetime	5.5 (0.2)
Mean (SD) substances used in past 3-months	4.4 (2.3)

### Sample substance use

The number of participants using each substance, and the frequency of use in the past 3-months is outlined in Table 2. Majority of participants reported lifetime use of tobacco (95.0%), cannabis (94.1%) and/or alcohol (92.1%). Highest mean ASSIST-Y Substance Specific Involvement (SSI) scores were reported among those using cannabis (21.4, SD = 10.4). Polysubstance use was common, with nearly 95% of our sample having used two-or more substances within their lifetime and 88.1% currently using two-or-more. The modal average number of substances was four (*n* = 20 lifetime, *n* = 16 current). Majority of participants (95%) had used all three of alcohol, tobacco and cannabis in their lifetime.

**Table 2:** Prevalence of self-reported substance use among sample

	Used in lifetime		Frequency of past 3-month use					ASSIST-Y risk-rating		
	N (%)	ASSIST-Y *M* (*SD*)	Daily or almost daily	Weekly	Monthly	Once or twice	No past 3-month use	Low N (%)	Moderate N (%)	High N (%)
Tobacco	96 (95.0)	14.6 (5.4)	86 (89.6)	5 (5.2)	1 (1.0)	1 (1.0)	3 (3.1)	8 (7.9)	11 (10.9)	82 (81.2)
Alcohol	93 (92.1)	10.9 (9.3)	12 (12.9)	31 (33.3)	18 (19.4)	17 (18.3)	15 (16.1)	43 (42.6)	32 (31.7)	26 (25.7)
Cannabis	95 (94.1)	21.4 (10.4)	74 (77.9)	7 (7.4)	1 (1.1)	6 (6.3)	7 (7.4)	12 (11.9)	12 (11.9)	77 (76.2)
Cocaine	50 (49.5)	12.0 (11.0)	9 (18.0)	17 (34.0)	3 (6.0)	11 (22.0)	10 (20.0)	56 (55.4)	16 (15.8)	29 (28.7)
Stimulants	51 (50.5)	9.9 (8.8)	11 (21.6)	12 (23.5)	7 (13.7)	8 (15.7)	13 (25.5)	58 (57.4)	16 (15.8)	27 (26.7)
Inhalants	37 (36.6)	16.3 (11.7)	1 (2.7)	3 (8.1)	2 (5.4)	6 (16.2)	25 (67.6)	78 (77.2)	18 (17.8)	5 (5.0)
Sedatives	57 (56.4)	4.1 (5.2)	19 (33.3)	13 (22.8)	6 (10.5)	10 (17.5)	9 (15.8)	52 (51.5)	10 (9.9)	39 (38.6)
Hallucinogens	31 (30.7)	3.6 (5.2)	2 (6.5)	2 (6.5)	2 (6.5)	8 (6.5)	17 (54.8)	81 (80.2)	14 (13.9)	6 (5.9)
Opioids	17 (16.8)	5.7 (6.6)	1 (5.9)	2 (11.8)	1 (5.9)	5 (29.4)	8 (47.1)	86 (85.1)	8 (7.9)	7 (6.9)
Other substances	31 (30.7)	14.8 (10.9)	10 (32.3)	8 (25.8)	3 (9.7)	6 (19.4)	4 (12.9)	73 (72.3)	8 (7.9)	20 (19.8)

### Primary outcomes

#### Internal consistency reliability

Table 3 summarizes reliability and agreement coefficients for ASSIST-Y for each substance. Bootstrapped Cronbach’s alpha coefficients (*n* = 1000 samples) indicated excellent reliability of the instrument for cocaine, and inhalants (α > 0.90); good reliability for alcohol, cannabis, and stimulants (α > 0.80), and acceptable reliability for opioids (α = 0.72) and sedatives (α = 0.68). Reliability was unable to be established for tobacco and hallucinogens (α = 0.59-0.60).

**Table 3:** Reliability coefficients for ASSIST-Y

	Internal Consistency		Cross-method agreement (moderate-risk vs. abuse)^*^					Cross-method agreement (high-risk vs dependence)				
	α^a^	95% CI	N	κ	S.E	95% CI	*p*	N	κ	S.E	95% CI	*p*
Tobacco	0.60	[0.43, 0.69]	-	-	-	-	-	-	-	-	-	-
Alcohol	0.81	[0.75, 0.86]	62	0.40	0.12	[0.18, 0.58]	0.001	77	0.46	0.15	[0.16, 0.62]	< 0.001
Cannabis	0.86	[0.80, 0.90]	17	0.29	0.15	[0.00, 0.29]	0.091	76	0.68	0.09	[0.50, 1.00]	< 0.001
Cocaine	0.90	[0.85, 0.94]	51	0.45	0.13	[0.20, 0.65]	< 0.001	76	0.13	0.09	[0.00, 0.09]	0.066
Stimulants	0.82	[0.74, 0.88]	58	0.53	0.13	[0.28, 0.81]	< 0.001	77	0.13	0.10	[0.00, 0.06]	0.071
Inhalants	0.93	[0.90, 0.95]	61	0.00	0.10	[0.00, -0.19]	0.984	76	0.08	0.10	[0.00, -0.04]	0.243
Sedatives	0.68	[0.47, 0.78]	49	0.52	0.14	[0.26, 0.78]	< 0.001	76	0.53	0.11	[0.32, 0.85]	< 0.001
Hallucinogens	0.59	[0.21, 0.74]	58	b	-	-	-	76	-0.07	0.04	[0.00, -0.21]	0.381
Opioids	0.72	[0.33, 0.85]	64	c	-	-	-	76	d	-	-	-

#### Cross-method agreement

Among those diagnosed with substance abuse (excluding cases of dependence), Kappa coefficients revealed acceptable agreement (κ ≥ 0.40) between the interview and ASSIST-Y risk outcomes for alcohol, cocaine, stimulants and sedatives (*p* ≤ .001). Agreement was questionable for cannabis (*p* = .091) and inhalants (*p* = .984). Insufficient case numbers were available to establish agreement for hallucinogens or opioids misuse. Among those with dependence, Kappa coefficients revealed good agreement between the interview and ASSIST-Y risk outcomes for cannabis (κ = 0.68, *p* < .001); acceptable agreement for sedatives (κ = 0.53, *p* < .001) and alcohol (κ = 0.46, *p* < .001); and questionable agreement for the remaining substances (see Table 3).

#### Concurrent validity

**Fig. 1:**
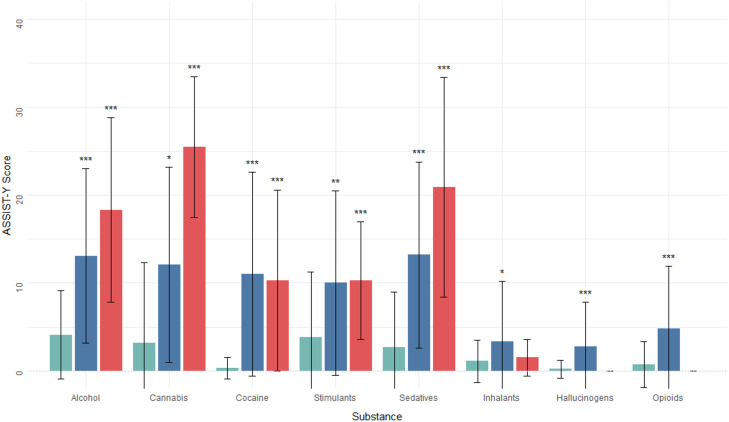
Distribution of ASSIST-Y substance specific involvement (SSI) scores by substance type, grouped by presence of MINI-plus DSM-IV substance use disorder (current). The chartdisplays the mean ASSIST-Y scores for each substance, divided into three categories: *No Current Disorder*, Current DSM-IV Abuse, and Current DSM-IV Dependence. The height of each barrepresents the mean SSI score for that group, while the whiskers indicate two standard deviations either side. The asterisks above the bars indicate the results of the convergent validityassessments (paired samples t-tests), comparing those in the No Current Disorder category to the other two categories, with one asterisk (*) indicating a significant difference between groupsat the *p < *0.05 level, two asterisks (**) indicating *p <* 0.01, and three asterisks (***) indicating *p <* 0.001

**Fig. 2:**
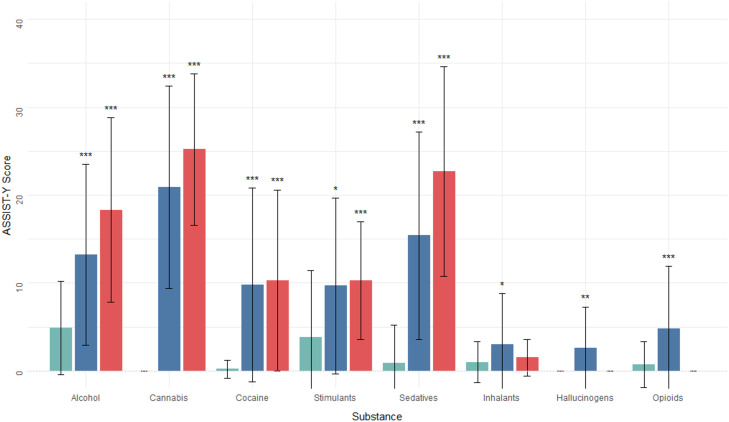
Distribution of ASSIST-Y substance specific involvement (SSI) scores by substance type, grouped by presence of MINI-plus DSM-IV substance use disorder (lifetime). The chartdisplays the mean ASSIST-Y scores for each substance, divided into three categories: No Lifetime Disorder, Lifetime DSM-IV Abuse, and Lifetime DSM-IV Dependence. The height of eachbar represents the mean SSI score for that group, while the whiskers indicate two standard deviations either side. The asterisks above the bars indicate the results of the convergent validityassessments (paired samples t-tests), comparing those in the No Lifetime Disorder category to the other two categories, with one asterisk (*) indicating a significant difference betweengroups at the *p <* 0.05 level, two asterisks (**) indicating *p <* 0.01, and three asterisks (***) indicating *p <* 0.001

Figure 1 displays ASSIST-Y SSI score distributions for each substance, grouped by current clinical diagnosis (based on the MINI-Plus categories). Similarly, Fig. 2 displays ASSIST-Y SSI score distributions, grouped by diagnosis of lifetime disorder. The figures show the concurrent validity of ASSIST-Y with respect to the diagnostic interview, through significantly higher ASSIST-Y scores among those with a diagnosis compared to those without. Specifically, for both lifetime and current disorders, scores were significantly higher for alcohol, cannabis, cocaine, stimulants and sedatives. Although higher scores were also found for the remaining substances, these differences were not statistically significant. A full summary of the pairwise t-test comparisons can be found in supplementary Table S2.

#### Convergent validity

ASSIST-Y cannabis scores demonstrated excellent convergent validity with scores on CAST (*r* = .85, *p* < .001), and good convergent validity with scores on SDS (*r* = .56, *p* < .001) and CRAFFT (*r* = .68, *p* < .001). ASSIST-Y tobacco scores demonstrated good convergent validity with SDS scores (*r* = .41, *p* < .001). Alcohol, cocaine, stimulants and sedatives also demonstrated good convergent validity with SDS (*r* = .36-0.55, *p* < .001) and CRAFFT (*r* = .31-0.47, *p* < .001). Convergent validity between ASSIST-Y SSIs and scores on SDS were also found to be good for inhalants (*r* = .32, *p* < .001) and opioids (*r* = .28, *p* = .005). Questionable convergent validity was identified between SSIs for hallucinogens and opioids and scores on CRAFFT, and between hallucinogens and scores on SDS (see supplementary Table S3 for a full correlation matrix).

### Secondary outcomes

#### Diagnostic accuracy

Table 4 presents a summary of diagnostic accuracy indices, with suggested cut-off scores for the ASSIST-Y based on MINI-Plus categorizations. Results suggested that differences in mean scores were significant at both the low vs. moderate-risk level, and the moderate vs. high-risk level for all substances except hallucinogens and opioids. ASSIST-Y was acceptable (i.e., AUC ≥ 0.70) in differentiating between low and moderate use for tobacco (Se. 94.9%; Sp. 28.9%), alcohol (Se. 69.0%; Sp. 80.0%), cannabis (Se. 88.9%; Sp. 100%), cocaine (Se. 75.0%; Sp. 97.2%), stimulants (Se. 75.0%; Sp. 73.6%), sedatives (Se. 71.6%; Sp. 91.4%), hallucinogens (Se. 40.5%; Sp. 100%) and opioids (Se. 50.0%; Sp. 91.9%). ASSIST-Y was also acceptable in differentiating between moderate and high-risk use for alcohol (Se. 55.6%; Sp. 92.6%), cannabis (Se. 94.2%; Sp. 70.8%), cocaine (Se. 75.0%; Sp. 69.4%), stimulants (Se. 66.7%; Sp. 78.4%), and sedatives (Se. 81.3%; Sp. 81.7%). AUC for moderate/high-risk tobacco and inhalant use were not found to be acceptable (AUC = 0.60-0.62), while no high-risk cases of opioids or hallucinogens were available for comparison.

**Table 4:** Diagnostic accuracy indices with optimal cut-off thresholds, and post-hoc comparisons for between group differences for each ASSIST-Y substance

	Diagnostic accuracy measures											Post-hoc tests	
	AUC (95% CI)	Se. (%)	Sp. (%)	PPV	NPV	LR +	LR-	CUI +	CUI-	*J*	Cut off	Z	*p*
Tobacco													
Low vs. moderate (ex. high)	0.60 (0.47, 0.72)	94.9	28.9	57.8	84.6	1.33	0.18	54.8	24.5	0.24	6.5	-6.4	< 0.001
(low/moderate) vs. high	0.61 (0.45, 0.76)	60.0	58.1	25.7	85.7	1.43	0.69	15.4	49.8	0.18	13.5	-8.3	< 0.001
Low vs. (moderate/high)	0.59 (0.46, 0.72)	93.3	19.4	21.9	92.3	1.16	0.35	20.4	17.9	0.24	6.5		
Alcohol													
Low vs. moderate (ex. high)	0.78 (0.68, 0.88)	69.0	80.0	80.6	68.3	3.45	0.39	55.6	54.6	0.49	6.5	-8.1	< 0.001
(low/moderate) vs. high	0.78 (0.59, 0.96)	55.6	92.6	50.0	94.0	7.51	0.48	27.8	87.1	0.48	22.5	0.5.5	< 0.001
Low vs. (moderate/high)	0.76 (0.65, 0.87)	66.7	75.7	74.3	68.3	2.74	0.44	49.5	51.7	0.48	8.0		
Cannabis													
Low vs. moderate (ex. high)	0.94 (0.89, 0.99)	88.9	100.0	100.0	33.3	*ind*	0.11	88.9	33.3	0.89	1.0	-4.3	< 0.001
(low/moderate) vs. high	0.87 (0.78, 0.97)	94.2	70.8	87.5	85.0	3.23	0.08	82.5	60.2	0.65	10.5	-5.2	< 0.001
Low vs. (moderate/high)	0.94 (0.89, 0.99)	87.5	100.0	100.0	33.3	*ind*	0.13	87.5	33.3	0.89	1.0		
Cocaine													
Low vs. moderate (ex. high)	0.86 (0.76, 0.95)	75.0	97.2	96.8	77.8	26.79	0.26	72.6	75.6	0.72	1.0	-7.9	< 0.001
(low/moderate) vs. high	0.70 (0.44, 0.97)	75.0	69.4	12.0	98.0	2.45	0.36	9.0	68.1	0.44	2.5	-4.2	< 0.001
Low vs. (moderate/high)	0.86 (0.77, 0.95)	75.0	97.2	96.8	77.8	26.79	0.26	72.6	75.6	0.72	1.0		
Stimulants													
Low vs. moderate (ex. high)	0.72 (0.60, 0.85)	75.0	73.6	56.3	86.7	2.84	0.34	42.2	63.8	0.49	1.0	-8.7	< 0.001
(low/moderate) vs. high	0.76 (0.63, 0.89)	66.7	78.4	11.1	98.3	3.09	0.42	7.4	77.0	0.45	11.0	-5.6	< 0.001
Low vs. (moderate/high)	0.72 (0.59, 0.84)	75.0	73.6	56.3	86.7	2.84	0.34	42.2	63.8	0.49	1.0		
Sedatives													
Low vs. moderate (ex. high)	0.85 (0.76, 0.94)	75.6	91.4	91.2	76.2	8.79	0.27	68.9	69.7	0.67	1.0	-8.0	< 0.001
(low/moderate) vs. high	0.82 (0.69, 0.96)	81.3	81.7	54.2	94.2	4.44	0.23	44.0	77.0	0.63	13.5	-5.4	< 0.001
Low vs. (moderate/high)	0.85 (0.76, 0.94)	75.6	91.4	91.2	76.2	8.79	0.27	68.9	69.7	0.68	5.5		
Inhalants													
Low vs. moderate (ex. high)	0.59 (0.44, 0.74)	34.8	81.1	44.4	74.1	1.84	0.80	15.5	60.1	0.16	1.0	-9.0	< 0.001
(low/moderate) vs. high	0.62 (0.22, 0.99)	50.0	82.4	7.1	98.4	2.84	0.61	3.6	81.1	0.32	2.5	-3.1	0.002
Low vs. (moderate/high)	0.59 9.44, 0.74)	34.8	81.1	44.4	74.1	1.84	0.80	15.5	60.1	0.17	2.5		
Hallucinogens													
Low vs. moderate (ex. high)	0.70 (0.58, 0.82)	40.5	100.0	100.0	63.9	*ind*	0.60	40.5	63.9	0.41	1.0	-	-
(low/moderate) vs. high	a	-	79.5	n/a	95.1	n/a	n/a	n/a	75.5	n/a	n/a	-	-
Low vs. (moderate/high)	0.70 (0.58, 0.82)	40.5	100.0	100.0	63.9	*ind*	0.60	40.5	63.9	0.41	1.0		
Opioids													
Low vs. moderate (ex. high)	0.70 (0.53, 0.87)	50.0	91.9	58.3	89.1	6.17	0.54	29.2	81.9	0.42	1.0	-	-
(low/moderate) vs. high	b	n/a	84.2	n/a	100.0	n/a	n/a	n/a	84.2	n/a	n/a	-	-
Low vs. (moderate/high)	0.70 (0.53, 0.87)	50.0	91.9	58.3	89.1	6.17	0.54	29.2	81.9	0.42	1.0		

#### Relative diagnostic performance.

 The relative performance of ASSIST-Y against other the other index screening tools (SDS, CRAFFT and CAST) was also assessed. AUC values for ASSIST-Y were highest among the assessments for alcohol, cocaine, stimulants and sedatives. For all instruments, AUC values on the other subscales were below the recommended level. ASSIST-Y also had the highest case-finding utility (CUI+) and screening utility (CUI-) for alcohol, cocaine, stimulants sedatives, inhalants among the assessments, while also having the highest screening utility (CUI-) for tobacco and hallucinogens. CAST was found to have higher clinical utility indices relative to ASSIST-Y for cannabis. Screening utility (CUI-) was found to be adequate-or-better across all substances (see Supplementary Table S4).

**Synthesis of results**. Table 5 summarizes the final outcomes from each assessment.

**Table 5:** Summary of reliability, validity and clinical utility assessment results

	Reliability/Agreement			Validity		Clinical Utility		
	Internal consistency^*^	Cross-method (abuse)	Cross-method (dependence)	Concurrent (abuse)	Concurrent (dependence)	Convergent	Case-finding	Screening
Tobacco	*Questionable*	Not assessed	Not assessed	Not assessed	Not assessed	Good	Acceptable	Acceptable
Alcohol	Good	Good	Acceptable	Excellent	Excellent	Good	Acceptable	Excellent
Cannabis	Good	*Questionable*	Good	Acceptable^b^	Excellent	Excellent	Excellent	Good
Cocaine	Excellent	Good	*Questionable*	Excellent	Good	Good	Good	Good
Stimulants	Good	Good	*Questionable*	Good	Good	Good	Acceptable	Good
Inhalants	Excellent	*Questionable*	*Questionable*	Good	*Questionable*	*Questionable*	Good	Excellent
Sedatives	*Questionable*	Good	Good	Excellent	Excellent	Good	*Questionable*	Good
Hallucinogens	*Questionable*	c	*Questionable*	Good	*Questionable*	*Questionable*	Acceptable	Good
Opioids	Acceptable	c	c	Excellent	c	Acceptable	*Questionable*	Excellent

*Did not meet assumptions of minimal adequate sample size (see Supplementary Table S1)

## Discussion

This study compared scores on ASSIST-Y, and corresponding risk stratifications to outcomes from a diagnostic interview and other standardized measures of substance-related harm and dependence. These properties were assessed among a sample of detained male adolescents in Barcelona, Spain. Results showed the ASSIST-Y performed well across a range of measures of validity, reliability and clinical utility.

The ASSIST-Y demonstrated good internal consistency reliability in general; though indices were not supported for hallucinogens and sedatives subscales. This may be due to the limited sample size overall (*n* = 31) who reported using hallucinogens, and the multivariate motivations driving hallucinogens use (as opposed to opioids for example). Moreover, it has been suggested that at least 10 individuals are required per test-item for Cronbach’s alpha [45], which means reliability estimates for inhalants, hallucinogens and opioids were likely underpowered (i.e., α < 0.50).

When compared to the clinical interview, the cross-method agreement between risk determinations and diagnostic outcomes was also mostly supported. Agreement indices comparing moderate-risk use (defined by ASSIST-Y) to DSM-IV abuse (defined by the diagnostic interview) were significant for alcohol, cocaine, stimulants and sedatives SSIs. Similarly, cross-method agreement between higher-risk use and DSM-IV dependence was significant for alcohol, cannabis and sedatives SSIs. The remaining substances were either not significant or could not be assessed due to limited sample size. Modifications to cut-off scores for ASSIST-Y is likely warranted, but further investigation with a larger, more representative sample is necessary.

In addition to risk categories, the total score distributions for each SSI were also significantly higher among those with a clinical diagnosis than those without. Although alignment with the interview classifications was not perfect for all substances, ASSIST-Y was able to differentiate between levels of DSM-IV-TR substance abuse well for all substances included in the analysis (i.e., those except tobacco). As a screening tool for detecting cases that warrant further investigation, this outcome is important. Moreover, most subscales also performed well when comparing higher-risk use vs. clinical dependence. While there were areas of misalignment, which is to be expected in a screening tool; the only subscales where concurrent validity could not be determined, were for inhalants, hallucinogens and opioids. Once more, this outcome is most likely due to the small number of participants engaged in high-risk use of these substances.

This study also assessed the convergent validity of ASSIST-Y relative to other standardized instruments. ASSIST-Y SSI scores were significantly associated with total scores on the other instruments, demonstrating good convergent validity for most substances. Again, insufficient cases of inhalants, hallucinogens, and opioids meant convergent validity for these substances could not be established.

The study also assessed the diagnostic accuracy, and relative performance of ASSIST-Y against the other standardized instruments, and found it significantly outperformed all other measures in identifying cases of harm, and ruling out cases of non-dependence (except in the unique case for cannabis versus CAST). These findings provide support for the use of ASSIST-Y in clinical and research settings, including for use in identifying adolescents who may be at risk of harm or dependence; particularly in the case of two or more co-occurring substances.

### Limitations

While this study has a number of strengths, it is important to acknowledge its limitations. Firstly, given the youth detention centre houses only young males, our sample did not include representation from other genders. Gender differences in the frequency and type of substances used, and harms associated are well documented [46], and therefore we must be careful not to generalize the results beyond our sample. Additional research is needed to investigate the psychometric properties of the instrument among females and other gender groups.

Second, the prevalence of substance use within the cohort of adolescents detained were significantly higher than would be expected in general health or medical settings. This intentional sampling decision was made to prioritize the inclusion of adolescents with higher levels of substance use, which may not reflect patterns of use (and therefore harm) of the broader adolescent population. However, the inclusion of non-substance-related detention would have necessitated a larger sample, and therefore would have required additional clinical resources to achieve the same result.

Third, the use of objective clinical markers, such as urine drug screens or other biological measures were not used to provide additional measures of validity. The reliance on clinical interviews using the MINI-Plus (FTND), are still subject to self-report biases. However, previous studies have demonstrated that self-disclosure measures of substance use are reliable (and in some cases may be more reliable than objective measures), provided there are no perceived adverse consequences [47, 48]. Relatedly, given a large proportion of the sample reported polysubstance use, it is difficult to determine the extent to which participants were able to accurately report on the specific harms associated with a particular substance versus another.

Fourth, the interview was based on DSM-IV classifications. Given updates to the new DSM-5 and ICD-11 criteria for substance-related disorders and dependence, future research should investigate validity and reliability of these instruments against the current classifications.

Finally, the ASSIST-Y is not structured to include questions around e-cigarette use/vaping. Given the increasing popularity of these products among youth, future research should be directed towards developing and validating a vaping SSI in the current instrument.

## Conclusions

As a screening questionnaire, the purpose of ASSIST-Y can identify adolescents and young people who may be at-risk of harm from substance use. While not designed for use as a diagnostic instrument, the ASSIST-Y was able to adequately differentiate between regular use, and use that might be consistent with dependence across the majority of commonly occurring substances. While the suggestion is not that ASSIST-Y should replace these tools in the clinical toolbox, ASSIST-Y provides an alternative to other instruments as it can quickly assess a wider variety of substances, as well as polysubstance use, and provide the basis for an intervention to reduce risk of harm, especially for non-specialist clinicians.

## Electronic supplementary material

Below is the link to the electronic supplementary material.

Supplementary Material 1

Supplementary Material 2

## Data Availability

The datasets generated and/or analysed during the current study are not publicly available due to the sensitive nature of the material and the age of participants, but may available from the corresponding author based upon reasonable request.

## Abbreviations

ASSIST: Alcohol, Smoking and Substance Involvement Screening Test
ASSIST-Y: Youth Alcohol, Smoking and Substance Involvement Screening Test (15–17 years)
AUC: Area Under the Curve
CAST: Cannabis Abuse Screening Test
CRAFFT: Car, Relax, Alone, Forget, Family or Friends Troubles
CUI: Clinical Utility Index
DSM: Diagnostic and Statistical Manual of Mental Disorders
FTND: Fagerstrӧm Test for Nicotine Dependence
ICD: International Classification of Diseases
LR: Likelihood Ratio
NPV: Negative predictive value
PPV: Positive predictive value
ROC: Receiver Operating Characteristics
SDS: Severity of Dependence Scale
SUD: Substance use disorder
SSIS: Substance Specific Involvement Score

## References

[1] Whiteford HA, Degenhardt L, Rehm J, Baxter AJ, Ferrari AJ, Erskine HE, Charlson FJ, Norman RE, Flaxman AD, Johns N, Burstein R. Global burden of disease attributable to mental and substance use disorders: findings from the global burden of Disease Study 2010. Lancet. 2013;382(9904):1575–86. 23993280 10.1016/S0140-6736(13)61611-6

[2] Squeglia LM, Jacobus J, Tapert SF. The influence of substance use on adolescent brain development. Clin EEG Neurosci. 2009;40(1):31–8. 19278130 10.1177/155005940904000110 PMC2827693

[3] Hall WD, Patton G, Stockings E, Weier M, Lynskey M, Morley KI, Degenhardt L. Why young people’s substance use matters for global health. Lancet Psychiatry. 2016;3(3):265–79. 26905482 10.1016/S2215-0366(16)00013-4

[4] Lamps CA, Sood AB, Sood R. Youth with substance abuse and comorbid mental health disorders. Curr Psychiatry Rep. 2008;10(3):265–71. 18652796 10.1007/s11920-008-0043-0

[5] Hamidullah S, Thorpe HH, Frie JA, Mccurdy RD, Khokhar JY. Adolescent substance use and the brain: behavioral, cognitive and neuroimaging correlates. Front Hum Neurosci. 2020;14:517606. 10.3389/fnhum.2020.00298 PMC7418456 32848673

[6] Gomes ST, Frota MV, Aguiar MB, Nogueira MB. Substance abuse and depression in adolescents. Neuropsychiatrie De l’Enfance et de l’Adolescence. 2012;5(60):S243.

[7] Quinn K, Walsh JL, Dickson-Gomez J. Multiple marginality and the variation in delinquency and substance use among adolescent gang members. Subst Use Misuse. 2019;54(4):612–27. 30395769 10.1080/10826084.2018.1528465 PMC6443478

[8] Chambers RA, Taylor JR, Potenza MN. Developmental neurocircuitry of motivation in adolescence: a critical period of addiction vulnerability. Am J Psychiatry. 2003;160(6):1041–52. 12777258 10.1176/appi.ajp.160.6.1041 PMC2919168

[9] Jordan CJ, Andersen SL. Sensitive periods of substance abuse: early risk for the transition to dependence. Dev Cogn Neurosci. 2017;25:29–44. 27840157 10.1016/j.dcn.2016.10.004 PMC5410194

[10] Halladay J, Woock R, El-Khechen H, Munn C, MacKillop J, Amlung M, Ogrodnik M, Favotto L, Aryal K, Noori A, Kiflen M. Patterns of substance use among adolescents: a systematic review. Drug Alcohol Depend. 2020;216:108222. 32971420 10.1016/j.drugalcdep.2020.108222

[11] Blanco C, Flórez-Salamanca L, Secades‐Villa R, Wang S, Hasin DS. Predictors of initiation of nicotine, alcohol, cannabis, and cocaine use: results of the national epidemiologic survey on Alcohol and related conditions (NESARC). Am J Addictions. 2018;27(6):477–84. 10.1111/ajad.12764 30088294

[12] Substance Abuse and Mental Health Services Administration (SAMHSA). Results from the 2005 national survey on drug use and health: national findings. 2006.

[13] Degenhardt L, Chiu WT, Sampson N, Kessler RC, Anthony JC, Angermeyer M, Bruffaerts R, De Girolamo G, Gureje O, Huang Y, Karam A. Toward a global view of alcohol, tobacco, cannabis, and cocaine use: findings from the WHO World Mental Health Surveys. PLoS Med. 2008;5(7):e141. 18597549 10.1371/journal.pmed.0050141 PMC2443200

[14] Stockings E, Hall WD, Lynskey M, Morley KI, Reavley N, Strang J, Patton G, Degenhardt L. Prevention, early intervention, harm reduction, and treatment of substance use in young people. Lancet Psychiatry. 2016;3(3):280–96. 26905481 10.1016/S2215-0366(16)00002-X

[15] Swift W, Coffey C, Carlin JB, Degenhardt L, Patton GC. Adolescent cannabis users at 24 years: trajectories to regular weekly use and dependence in young adulthood. Addiction. 2008;103(8):1361–70. 18855826 10.1111/j.1360-0443.2008.02246.x

[16] Degenhardt L, Ferrari AJ, Calabria B, Hall WD, Norman RE, McGrath J, Flaxman AD, Engell RE, Freedman GD, Whiteford HA, Vos T. The global epidemiology and contribution of cannabis use and dependence to the global burden of disease: results from the GBD 2010 study. PLoS ONE. 2013;8(10):e76635. 24204649 10.1371/journal.pone.0076635 PMC3811989

[17] Degenhardt L, Baxter AJ, Lee YY, Hall W, Sara GE, Johns N, Flaxman A, Whiteford HA, Vos T. The global epidemiology and burden of psychostimulant dependence: findings from the global burden of Disease Study 2010. Drug Alcohol Depend. 2014;137:36–47. 24559607 10.1016/j.drugalcdep.2013.12.025

[18] Degenhardt L, Charlson F, Mathers B, Hall WD, Flaxman AD, Johns N, Vos T. The global epidemiology and burden of opioid dependence: results from the global burden of disease 2010 study. Addiction. 2014;109(8):1320–33. 24661272 10.1111/add.12551

[19] Degenhardt L, Stockings E, Patton G, Hall WD, Lynskey M. The increasing global health priority of substance use in young people. Lancet Psychiatry. 2016;3(3):251–64. 26905480 10.1016/S2215-0366(15)00508-8

[20] Hawkins EH. A tale of two systems: co-occurring mental health and substance abuse disorders treatment for adolescents. Ann Rev Psychol. 2009;60:197–227. 19035824 10.1146/annurev.psych.60.110707.163456

[21] Hadland SE, Yule AM, Levy SJ, Hallett E, Silverstein M, Bagley SM. Evidence-based treatment of young adults with substance use disorders. Pediatrics. 2021;147(Supplement 2):S204–14. 33386323 10.1542/peds.2020-023523D PMC7879425

[22] Volkow ND, Han B, Einstein EB, Compton WM. Prevalence of substance use disorders by time since first substance use among young people in the US. JAMA Pediatr. 2021;175(6):640–3. 33779715 10.1001/jamapediatrics.2020.6981 PMC8008418

[23] WHO-ASSIST Working Group. The alcohol, smoking and substance involvement screening test (ASSIST): development, reliability and feasibility. Addiction. 2002;97(9):1183–94. 12199834 10.1046/j.1360-0443.2002.00185.x

[24] Humeniuk R, Ali R, Babor TF, Farrell M, Formigoni ML, Jittiwutikarn J, De Lacerda RB, Ling W, Marsden J, Monteiro M, Nhiwatiwa S. Validation of the alcohol, smoking and substance involvement screening test (ASSIST). Addiction. 2008;103(6):1039–47. 18373724 10.1111/j.1360-0443.2007.02114.x

[25] Humeniuk R, Holmwood C, Beshara M, Kambala A. ASSIST-Y v1. 0: first-stage development of the who alcohol, smoking and substance involvement screening test (assist) and linked brief intervention for young people. J Child Adolesc Subst Abuse. 2016;25(4):384–90.

[26] Källmén H, Berman AH, Jayaram-Lindström N, Hammarberg A, Elgán TH. Psychometric properties of the AUDIT, AUDIT-C, CRAFFT and ASSIST-Y among Swedish adolescents. Eur Addict Res. 2019;25(2):68–77. 30726842 10.1159/000496741

[27] World Health Organization. Process of translation and adaptation of instruments. http://www.who.int/substance_abuse/research_tools/translation/en/.2009

[28] Legleye S, Karila L, Beck F, Reynaud M. Validation of the CAST, a general population Cannabis abuse screening test. J Subst use. 2007;12(4):233–42.

[29] Rial A, García-Couceiro N, Gómez P, Mallah N, Varela J, Flórez-Menéndez G, Isorna M. Psychometric properties of CAST for early detection of problematic cannabis use in Spanish adolescents. Addict Behav. 2022;129:107288. 35219995 10.1016/j.addbeh.2022.107288

[30] Fernandez-Artamendi S, Fernández-Hermida JR, Muñiz-Fernández J, Secades-Villa R, García-Fernández G. Screening of cannabis-related problems among youth: the CPQ-AS and CAST questionnaires. Subst Abuse Treat Prev Policy. 2012;7(1):1–0. 22471908 10.1186/1747-597X-7-13 PMC3375190

[31] Gossop M, Darke S, Griffiths P, Hando J, Powis B, Hall W, Strang J. The severity of dependence scale (SDS): psychometric properties of the SDS in English and Australian samples of heroin, cocaine and amphetamine users. Addiction. 1995;90(5):607–14. 7795497 10.1046/j.1360-0443.1995.9056072.x

[32] Lawrinson P, Copeland J, Gerber S, Gilmour S. Determining a cut-off on the severity of dependence scale (SDS) for alcohol dependence. Addict Behav. 2007;32(7):1474–9. 17081703 10.1016/j.addbeh.2006.09.005

[33] Cuevas CD, Sanz EJ, Fuente JA, Padilla J, Berenguer JC. The severity of dependence scale (SDS) as screening test for benzodiazepine dependence: SDS validation study. Addiction. 2000;95(2):245–50. 10723853 10.1046/j.1360-0443.2000.95224511.x

[34] Vélez-Moreno A, González-Saiz F, Rojas AJ, Torrico-Linares E, Fernández-Calderón F, Ramírez-López J, Lozano OM. Reliability and validity of the Spanish version of the substance dependence severity scale. Eur Addict Res. 2014;21(1):39–46. 25376716 10.1159/000365282

[35] Knight JR, Shrier LA, Bravender TD, Farrell M, Vander Bilt J, Shaffer HJ. A new brief screen for adolescent substance abuse. Arch Pediatr Adolesc Med. 1999;153(6):591–6. 10357299 10.1001/archpedi.153.6.591

[36] Rial A, Kim-Harris S, Knight JR, Araujo M, Gómez P, Braña T, Varela J, Golpe S. Empirical validation of the CRAFFT abuse screening test in a Spanish sample. Validación empírica del CRAFFT abuse screening. Test en una muestra de adolescentes españoles. Adicciones. 2019;31:160–9. 29353300 10.20882/adicciones.1105

[37] Heatherton TF, Kozlowski LT, Frecker RC, FAGERSTROM KO. The Fagerström test for nicotine dependence: a revision of the Fagerstrom Tolerance Questionnaire. Br J Addict. 1991;86(9):1119–27. 1932883 10.1111/j.1360-0443.1991.tb01879.x

[38] Sheehan DV, Lecrubier Y, Sheehan KH, Amorim P, Janavs J, Weiller E, Hergueta T, Baker R, Dunbar GC. The mini-international neuropsychiatric interview (MINI): the development and validation of a structured diagnostic psychiatric interview for DSM-IV and ICD-10. J Clin Psychiatry. 1998;59(20):22–33. 9881538

[39] Ferrando L, Franco AL, Soto M, Bobes J, Soto O, Franco L, Gubert J. MINI international neuropsychiatric interview. Spanish version 5.0. 0. DSM-IV.

[40] RStudio T, RStudio. PBC, Boston, MA URL http://www.rstudio.com/.

[41] Viera AJ, Garrett JM. Understanding interobserver agreement: the kappa statistic. Fam med. 2005;37(5):360–3. 15883903

[42] Cohen J. Statistical power analysis for the behavioral sciences. Volume 3. Academic; 2013 Sep.

[43] Hedges LV. Distribution theory for Glass’s estimator of effect size and related estimators. J Educational Stat. 1981;6(2):107–28.

[44] Cohen J. A power primer. 2016.

[45] Bonett DG. Sample size requirements for testing and estimating coefficient alpha. J Educational Behav Stat. 2002;27(4):335–40.

[46] Kloos A, Weller RA, Chan R, Weller EB. Gender differences in adolescent substance abuse. Curr Psychiatry Rep. 2009;11(2):120–6. 19302765 10.1007/s11920-009-0019-8

[47] Babor TF, Steinberg K, Anton RA, Del Boca F. Talk is cheap: measuring drinking outcomes in clinical trials. J Stud Alcohol. 2000;61(1):55–63. 10627097 10.15288/jsa.2000.61.55

[48] Del Boca FK, Noll JA. Truth or consequences: the validity of self-report data in health services research on addictions. Addiction. 2000;95(11s3):347–60. 11132362 10.1080/09652140020004278

