# Supplementary material for: Validity, reliability and clinical utility of ASSIST-Y in assessing risk of substance-related harm and dependence in Spanish male adolescents
Source: Child Adolesc Psychiatry Ment Health. 2025 Jan 13;19:1. doi: 10.1186/s13034-024-00845-6 (PMC11731557; doi:10.1186/s13034-024-00845-6)
Supplement: Supplementary file 1 — Supplementary Material 1 [file 13034_2024_845_MOESM1_ESM.docx]

## Statistical approach

### Sample size and power calculations

In order for the analyses to be adequately powered (β=.90), with a type-II error rate of 5% or less (α=.05), we calculated minimum sample sizes required for each analysis. These are discussed in the sections that follow.

## Reliability

### Internal consistency reliability

Cronbach’s alpha is widely used to assess internal consistency reliability [1]. However, a more correct interpretation is that the test actually assesses the uni-dimensionality of a set of test items. Too few, or too many items can cause Cronbach’s alpha values to be biased downwards [2]. Theoretically, provided each item in the scale assesses the same unidimensional construct, Cronbach’s alpha coefficients should be meaningful. Since subscales for alcohol and each of the illicit substances included 5-items; which, we hypothesise were uni-dimensionally-related to SUD-risk severity, we assume that Cronbach’s alpha will be a reliable indicator of each subscale’s internal consistency. However, since our tobacco subscale only contains four items, there is the potential that too few scale items would lead to inaccurate results. Therefore, we decided to use bootstrapping to approximate the alpha coefficient, with 1000 samples. This calculated a distribution of Cronbach’s alpha coefficients which we could reasonably expect from our sample. Moreover, since the scale items use previously-defined score weightings that are not consistent across items (i.e., some items are scored between 0 and 5, while some are scored between 0 and 8), it was necessary to standardize score items. We used min-max scaling, whereby for each given individual score on each given item, we subtracted the minimum possible score from each individual’s score, and divided by the score range. This re-scaled each score item to range between 0 and 1 for each item, allowing us to compare adequately without biased estimates.

We calculated the minimum sample size required to detect a Cronbach’s alpha of .70 or greater, setting the alpha significance to .05, with a minimum power of .90, with the number of scale items equal to 4 (tobacco) or 5 (all other substances). This method has been suggested previously [3,4], and has been used widely in health and medical research since [5]. The result was a minimum sample size of 38 for a 5-item scale, and 40 for the 4-item scale. We elected to use 40 as the minimum sample size for consistency.

### Cross-method agreement

We assessed the cross-method agreement of ASSIST-Y risk outcomes with the MINI-Plus diagnoses using Cohen’s Kappa. For each outcome, we created a 2x2 matrix, with ASSIST-Y classification in the rows, and MINI-Plus diagnoses as the columns. To assess cross-method agreement between moderate-risk ASSIST ratings with MINI-Plus abuse ratings, we included all instances of low-risk (coded 0) and moderate-risk (coded 1), and MINI-Plus non-abuse (0) and abuse (1). Since substance use disorders exist on a continuum, from non-problematic use to dependence, with ‘abuse’ falling somewhere along the spectrum; and likewise ASSIST-Y classifications existing on a continuum from low to high, with moderate-risk falling in the middle; it was necessary to remove all instances of higher risk use or dependence from the comparison of abuse/moderate risk. This prevented the co-mingling of low- and high-risk groups in the analysis. Comparisons of high-risk use with MINI-Plus dependence included the entire sample. For both sets of analyses, we reported Cohen’s Kappa, with 95% confidence intervals and tests of significance. We followed guidelines set out by Viera & Garret [6] to determine the strength of agreement.

We calculated the minimum sample size required to achieve a kappa value of .60 or more, at an alpha level of .05, and power of .10, and a disagreement rate of .95. We used Temel and Erdogan’s [7] method for calculating the sample size, and determined the value to be 73 participants.

# Validity

### Concurrent validity

To assess the concurrent validity of the ASSIST-Y with the gold-standard clinical interview, we also compared mean substance specific involvement (SSI) scores between groups based on the presence or absence of diagnosis for either lifetime or current abuse or dependence. We used two-tailed paired-samples t-tests (with 95% confidence intervals), reporting Hedge’s *g* [8] as the measure of effect size, using Cohen’s standards to determine small, medium and large effect [9]. Significant effect sizes greater than .20 were deemed to indicate acceptable concurrent validity. Minimum required sample size was determined based on an effect size of .30 or greater, at an alpha level of .05 and power of .80. The minimum sample size was determined to be 90 participants.

### Convergent validity

We assessed convergent validity through a correlation between ASSIST-Y SSI scores against SDS and CRAFFT; with an additional assessment of SSI cannabis scores against CAST. As CRAFFT does not assess tobacco, we focused on illicit substances and alcohol. A Pearson’s *R* correlation matrix was used to assess the strength and direction of correlations. Cohen’s [10] standards were used to determine weak (.20-.49), moderate (.50-.79), and strong (>.80) coefficients. Significant correlations greater than .20 were deemed to indicate acceptable convergent validity.

## Clinical Utility

To achieve an adequately powered AUC equal to .70 or higher, with a ratio of 1:1 (negative/positive cases [based on ASSIST-Y scores]) a minimum of 31 participants are needed for each group. Fewer participants would be required in the event that the ratio of negative/positive cases was higher.

Sensitivity describes the proportion of correctly identified cases of dependence among all those who are dependent in the sample (TP/TP+FN). Specificity describes the proportion of correctly identified cases of non-dependence among all those who are not dependent in the sample (TN/FP+TN). PPV describes the proportion of correctly identified cases among the total number of those who were indicated as being dependent (TP/TP+FP). NPV describes the proportion of correctly identified non-cases among the total of those who were indicated as not being dependent (TN/TN+FN). AUC describes the probability, if given one TP, that that TP will score higher than one TN on the test. The criterion for AUC will be set at ≥0.70, to indicate at least better than acceptable accuracy. LR+ describes the ratio of true cases of dependence to false cases, while LR- describes the likelihood of true non-cases of dependence to false non-cases. Higher ratios indicate better accuracy. CUI+ is the product of sensitivity and positive predictive value, while CUI- is the product of specificity and the negative predictive value.

# REFERNCES

1. Cronbach, L. J. (1951). Coefficient alpha and the internal structure of tests. *psychometrika*, *16*(3), 297-334.
2. Tavakol, M., & Dennick, R. (2011). Making sense of Cronbach's alpha. International journal of medical education, 2, 53–55. <https://doi.org/10.5116/ijme.4dfb.8dfd>
3. Bonett, D. G. (2002). Sample size requirements for testing and estimating coefficient alpha. Journal of educational and behavioral statistics, 27(4), 335-340.
4. Streiner, D. L. (2003). Starting at the beginning: an introduction to coefficient alpha and internal consistency. Journal of personality assessment, 80(1), 99-103. https://doi.org/10.21315/mjms2018.25.6.9
5. Bujang, M. A., Omar, E. D., & Baharum, N. A. (2018). A Review on Sample Size Determination for Cronbach's Alpha Test: A Simple Guide for Researchers. The Malaysian journal of medical sciences : MJMS, 25(6), 85–99. Lord, F. M., & Novick, M. R. (2008). Statistical theories of mental test scores. IAP.
6. Viera, A. J., & Garrett, J. M. (2005). Understanding interobserver agreement: the kappa statistic. Fam med, 37(5), 360-363.
7. Temel, G., & Erdogan, S. (2017). Determining the sample size in agreement studies. Marmara Medical Journal, 30(2), 101-112.
8. Hedges, L. V. (1981). Distribution Theory for Glass's Estimator of Effect size and Related Estimators. Journal of Educational Statistics, 6(2), 107-128.
9. Cohen J. A power primer. 2016.
10. Cohen J. Statistical power analysis for the behavioral sciences. Academic press; 2013 Sep 3.
11. Dhand, N. K., & Khatkar, M. S. (2014). Statulator: An online statistical calculator. Sample Size Calculator for Comparing Two Paired Means.
