# Supplementary material for: Validity, reliability and clinical utility of ASSIST-Y in assessing risk of substance-related harm and dependence in Spanish male adolescents
Source: Child Adolesc Psychiatry Ment Health. 2025 Jan 13;19:1. doi: 10.1186/s13034-024-00845-6 (PMC11731557; doi:10.1186/s13034-024-00845-6)
Supplement: Supplementary file 2 — Supplementary Material 2 [file 13034_2024_845_MOESM2_ESM.docx]

| **Table S1**. Coefficients of determination for analyses. | | | | | | | | |
| --- | --- | --- | --- | --- | --- | --- | --- | --- |
|  | Parameters | | |  | Interpretation | | | |
|  | Statistical test | Coefficient | Min sample size^a^ |  | Excellent | Good | Acceptable | *Questionable* |
| **Reliability** |  |  |  |  |  |  |  |  |
| Internal consistency | Internal consistency | Cronbach’s alpha (α) | 40 |  | ≥.90 | .80 - .89 | .70 - .79 | <.69 or non-sig. α |
| cross-method agreement | Cross-tabulated agreement | Cohen’s Kappa (κ) | 73 |  | .81 - .99 | .61 - .80 | .41 - .60 | < .40; or non-sig. κ |
| **Validity** |  |  |  |  |  |  |  |  |
| Concurrent | Paired-Samples t-test | Hedges’ g (*g*) | 90 |  | ≥.80 | .50 - .79 | .30 - .49 | <.30 or non-sig. *g* |
| Convergent | Pearson’s test of association | Pearson’s R (*r*) | 84 |  | ≥.60 | .40 - .59 | .20 - .40 | <.20 or no-sig. *r* |
| **Clinical Utility** |  |  |  |  |  |  |  |  |
| Case-finding utility | Sensitivity $\times$ PPV | CUI + | n/a |  | ≥.80 | .60 - .79 | .40 - .59 | <.40 |
| Screening utility | Specificity $\times$ NPV | CUI - | n/a |  | ≥.80 | .60 - .79 | .40 - .59 | <.40 |
| *Note*: a) Minimum sample size calculations discussed in each corresponding section of supplementary materials.  non-sig.= Not statistically significant (i.e. *p>*.05). PPV=Positive Predictive Value; NPV=Negative Predictive Value, CUI+=Positive Clinical Utility Index, CUI-=Negative Clinical Utility Index. | | | | | | | | |

| **Supplementary Table S2**. Pairwise t-tests between ASSIST-Y SSI scores with MINI-Plus diagnoses of lifetime or current abuse or dependence (versus no disorder). | | | | | | | | | |
| --- | --- | --- | --- | --- | --- | --- | --- | --- | --- |
|  | ASSIST-Y Substance Specific Inventory scores | | | |  |  | | | |
|  | MINI Diagnosis present | | MINI Diagnosis absent | |  | Test Statistics | | | |
|  | N (%) | *M* (*SD*) | N (%) | *M* (*SD*) |  | *t*-test | *p* | *g* | [95% CI] |
| Alcohol |  |  |  |  |  |  |  |  |  |
| Lifetime abuse | 31 (46.3) | 13.2 (10.3) | 36 (53.7) | 4.9 (5.3) |  | -4.2 | <.001 | -1.03 | [-1.53, -0.52] |
| Lifetime dependence | 9 (11.7) | 18.3 (10.5) | 68 (88.3) | 8.6 (9.0) |  | -3.0 | .002 | -1.05 | [-1.76, -0.34] |
| Current abuse | 34 (50.0) | 13.1 (9.9) | 34 (50.0) | 4.1 (5.0) |  | -4.7 | <.001 | -1.13 | [-1.63, -0.62] |
| Current dependence | 9 (11.7) | 18.3 (10.5) | 68 (88.3) | 8.6 (9.0) |  | -3.0 | .002 | -1.05 | [-1.76, -0.34] |
| Cannabis |  |  |  |  |  |  |  |  |  |
| Lifetime abuse | 73 (94.8) | 20.9 (11.5) | 4 (5.2) | .0 (0.0) |  | -3.6 | <.001 | -1.84 | [-2.87, -.80] |
| Lifetime dependence | 51 (66.2) | 25.2 (8.6) | 26 (33.8) | 9.3 (11.2) |  | -6.9 | <.001 | -1.65 | [-2.20, -1.10] |
| Current abuse | 16 (53.3) | 12.1 (11.1) | 10 (46.7) | 3.2 (9.1) |  | -2.1 | .044 | .06 | [-0.64, 0.76] |
| Current dependence | 51 (66.2) | 25.5 (8.0) | 26 (33.8) | 8.7 (11.1) |  | -7.6 | <.001 | -1.81 | [-2.36, -1.26] |
| Cocaine |  |  |  |  |  |  |  |  |  |
| Lifetime abuse | 41 (53.2) | 9.8 (11.0) | 36 (46.8) | .2 (1.0) |  | -5.2 | <.001 | -1.18 | [-1.65, -0.69] |
| Lifetime dependence | 4 (5.2) | 10.3 (10.3) | 73 (94.8) | 5.0 (9.3) |  | -1.1 | .277 | -.56 | [-0.16, 0.45] |
| Current abuse | 32 (43.2) | 11.0 (11.6) | 42 (56.8) | .3 (1.2) |  | .5.9 | <.001 | -1.38 | [-1.88, -0.87] |
| Current dependence | 4 (5.2) | 10.3 (10.3) | 73 (94.8) | 5.0 (9.3) |  | -1.1 | .277 | -.56 | [-0.16, 0.45] |
| Stimulants |  |  |  |  |  |  |  |  |  |
| Lifetime abuse | 25 (32.5) | 9.7 (10.0) | 52 (67.5) | 3.8 (7.6) |  | -2.9 | .005 | -.69 | [-1.17, -0.20] |
| Lifetime dependence | 3 (3.9) | 10.3 (6.7) | 74 (96.1) | 5.5 (8.9) |  | -.9 | .359 | -.54 | [-1.68, 0.61] |
| Current abuse | 21 (28.4) | 10.0 (10.5) | 53 (71.6) | 3.8 (7.5) |  | -2.9 | .005 | -.73 | [-1.24, -0.22] |
| Current dependence | 3 (3.9) | 10.3 (6.7) | 74 (96.1) | 5.5 (8.9) |  | -.9 | .359 | -.54 | [-1.68, 0.61] |
| Sedatives |  |  |  |  |  |  |  |  |  |
| Lifetime abuse | 41 (53.9) | 15.4 (11.8) | 35 (46.1) | .9 (4.3) |  | -6.9 | <.001 | -1.56 | [-2.07, -1.05] |
| Lifetime dependence | 14 (18.2) | 22.7 (11.9) | 63 (81.8) | 5.5 (8.9) |  | -6.1 | <.001 | -1.80 | [-2.40, -1.10] |
| Current abuse | 17 (27.9) | 13.2 (10.6) | 44 (72.1) | 2.7 (6.3) |  | -4.8 | <.001 | -1.34 | [-1.94, -0.74] |
| Current dependence | 16 (20.8) | 20.9 (12.5) | 61 (79.2) | 5.4 (9.0) |  | -5.6 | <.001 | -1.57 | [-2.17, -0.97] |
| Hallucinogens |  |  |  |  |  |  |  |  |  |
| Lifetime abuse | 38 (49.4) | 2.6 (4.7) | 39 (50.6) | .0 (0.0) |  | -3.4 | <.001 | -.78 | [-1.23, -0.32] |
| Lifetime dependence | 3 (3.9) | 0.0 (0.0) | 74 (96.1) | 1.3 (3.6) |  | .6 | .526 | .37 | [-0.77, 1.51] |
| Current abuse | 32 (42.7) | 2.8 (5.0) | 43 (57.3) | .2 (1.0) |  | -3.3 | .001 | -.76 | [-1.23, -0.29] |
| Current dependence | 3 (3.9) | 0.0 (0.0) | 74 (96.1) | 1.3 (3.6) |  | .6 | .526 | .37 | [-0.77, 1.51] |
| Inhalants |  |  |  |  |  |  |  |  |  |
| Lifetime abuse | 23 (29.9) | 3.0 (5.8) | 54 (70.1) | 1.0 (2.3) |  | -2.2 | .035 | -.53 | [-1.02, -0.04] |
| Lifetime dependence | 2 (2.6) | 1.5 (2.1) | 75 (97.4) | 1.6 (3.8) |  | .0 | .983 | .02 | [-1.38, 1.40] |
| Current abuse | 16 (21.3) | 3.3 (6.9) | 59 (78.7) | 1.1 (2.4) |  | -2.0 | .047 | -.56 | [-1.11, -0.01] |
| Current dependence | 2 (2.6) | 1.5 (2.1) | 75 (97.4) | 1.6 (3.8) |  | .0 | .983 | .02 | [-1.38, 1.40] |
| Opioids |  |  |  |  |  |  |  |  |  |
| Lifetime abuse | 14 (18.2) | 4.8 (7.1) | 63 (81.8) | .7 (2.6) |  | -3.7 | <.001 | -1.09 | [-1.69, -0.49] |
| Lifetime dependence | 0 (0) | 0 (0) | 77 (100) | 1.4 (4.0) |  | - | - | - | - |
| Current abuse | 14 (18.2) | 4.8 (7.1) | 62 (81.8) | .7 (2.6) |  | -3.7 | <.001 | -1.08 | [-1.68, -0.48] |
| Current dependence | 0 (0) | 0 (0) | 77 (100) | 1.4 (4.0) |  | - | - | - | - |
|  | | | | | | | | | |

| **Supplementary Table S3**. Total score convergent validity - Pearson’s R correlation matrix of associations between ASSIST-Y SSI scores, and total scores on CAST, SDS and CRAFFT. | | | | | | | | | | | | |
| --- | --- | --- | --- | --- | --- | --- | --- | --- | --- | --- | --- | --- |
|  | Tobacco | Alcohol | Cannabis | Cocaine | Stimulants | Inhalants | Sedatives | Hallucinogens | Opioids | Other | SDS | CAST |
| SSI Tobacco | -- |  |  |  |  |  |  |  |  |  |  |  |
| SSI Alcohol | **.44** | -- |  |  |  |  |  |  |  |  |  |  |
| SSI Cannabis | **.40** | **.32** | -- |  |  |  |  |  |  |  |  |  |
| SSI Cocaine | **.31** | **.46** | .18 | -- |  |  |  |  |  |  |  |  |
| SSI Stimulants | .18 | **.37** | **.21** | **.55** | -- |  |  |  |  |  |  |  |
| SSI Inhalants | .10 | **.24** | .14 | **.28** | **.47** | -- |  |  |  |  |  |  |
| SSI Sedatives | **.27** | **.25** | **.23** | **.59** | **.46** | **.34** | -- |  |  |  |  |  |
| SSI Hallucinogens | .14 | .15 | .13 | **.44** | **.37** | **.32** | **.21** | -- |  |  |  |  |
| SSI Opioids | .06 | .00 | .18 | .18 | .19 | .18 | **.29** | **.49** | -- |  |  |  |
| SSI Other | .07 | .12 | .15 | **.32** | **.33** | .08 | **.53** | **.22** | **.36** | -- |  |  |
| Total SDS | **.41** | **.36** | **.56** | **.40** | **.43** | **.32** | **.55** | .13 | **.28** | **.36** |  |  |
| Total CAST | **.36** | **.31** | **.85** | **.21** | **.35** | **.22** | **.37** | .14 | **.26** | **.29** | **.64** | -- |
| Total CRAFFT | **.54** | **.47** | **.68** | **.35** | **.31** | .16 | **.41** | .13 | .17 | **.24** | **.63** | **.72** |
| Note: SSI=Substance Specific Score; SDS=Severity of Dependence Scale; CAST=Cannabis Abuse Screening Test; CRAFFT=CRAAFT Screening Tool;  Bold values indicate *p*<.05 | | | | | | | | | | | | |

| **Supplementary Table S4**. Relative performance and clinical utility of ASSIST-Y versus other standardised measures in screening for cases for dependence. | | | | | | | | | |
| --- | --- | --- | --- | --- | --- | --- | --- | --- | --- |
|  | Diagnostic accuracy measures | | | | | | | | |
|  | AUC (95% CI) | Se. (%) | Sp. (%) | PPV | NPV | LR+ | LR- | CUI+ | CUI- |
| Tobacco |  |  |  |  |  |  |  |  |  |
| SDS | .63 (.48, .78) | 86.7 | 30.6 | 23.2 | 90.5 | 1.25 | .43 | 20.1 | 27.7 |
| ASSIST-Y | .61 (.45, .76) | 60.0 | 58.1 | 25.7 | 85.7 | 1.43 | .69 | 15.4 | 49.8 |
| Alcohol |  |  |  |  |  |  |  |  |  |
| SDS | .64 (.50, .83) | 88.9 | 29.4 | 14.3 | 95.2 | 1.26 | .38 | 12.7 | 28.0 |
| CRAFFT | .59 (.39, .78) | 88.9 | 17.6 | 12.5 | 92.3 | 1.08 | .63 | 11.1 | 16.3 |
| ASSIST-Y | .78 (.59, .96) | 55.6 | 92.6 | 50.0 | 94.0 | 7.51 | .48 | 27.8 | 87.1 |
| Cannabis |  |  |  |  |  |  |  |  |  |
| SDS | .81 (.71, .91) | 86.5 | 58.3 | 81.8 | 66.7 | 2.07 | .23 | 70.8 | 38.9 |
| CRAFFT | .79 (.70, .92) | 96.2 | 45.8 | 79.4 | 84.6 | 1.77 | .08 | 76.3 | 38.8 |
| CAST | .92 (.85, .98) | 94.2 | 75.0 | 89.1 | 85.7 | 3.77 | .08 | 84.0 | 64.3 |
| ASSIST-Y | .87 (.78, .97) | 94.2 | 70.8 | 87.5 | 85.0 | 3.23 | .08 | 82.5 | 60.2 |
| Cocaine |  |  |  |  |  |  |  |  |  |
| SDS | .51 (.31, .71) | 100.0 | 29.2 | 7.3 | 100.0 | 1.41 | .00 | 7.3 | 29.2 |
| CRAFFT | .64 (.44, .84) | 100.0 | 18.1 | 6.3 | 100.0 | 1.22 | .00 | 6.3 | 18.1 |
| ASSIST-Y | .70 (.44, .97) | 75.0 | 69.4 | 12.0 | 98.0 | 2.45 | .36 | 9.0 | 68.1 |
| Stimulants |  |  |  |  |  |  |  |  |  |
| SDS | .70 (.40, .99) | 100.0 | 28.4 | 5.4 | 100.0 | 1.40 | .00 | 5.4 | 28.4 |
| CRAFFT | .58 (.36, .80) | 100.0 | 17.6 | 4.7 | 100.0 | 1.21 | .00 | 4.7 | 17.6 |
| ASSIST-Y | .76 (.63, .89) | 66.7 | 78.4 | 11.1 | 98.3 | 3.09 | .42 | 7.4 | 77.0 |
| Sedatives |  |  |  |  |  |  |  |  |  |
| SDS | .72 (.58, .87) | 93.8 | 33.3 | 27.3 | 95.2 | 1.41 | .19 | 25.6 | 31.7 |
| CRAFFT | .58 (.44, .71) | 100.0 | 21.7 | 25.4 | 100.0 | 1.28 | .00 | 25.4 | 21.7 |
| ASSIST-Y | .82 (.69, .96) | 81.3 | 81.7 | 54.2 | 94.2 | 4.44 | .23 | 44.0 | 77.0 |
| Inhalants |  |  |  |  |  |  |  |  |  |
| SDS | .55 (.44, .67) | 100.0 | 28.4 | 3.6 | 100.0 | 1.40 | .00 | 3.6 | 28.4 |
| CRAFFT | .63 (.36, .91) | 100.0 | 17.6 | 3.2 | 100.0 | 1.21 | .00 | 3.2 | 17.6 |
| ASSIST-Y | .62 (.22, .99) | 50.0 | 82.4 | 7.1 | 98.4 | 2.84 | .61 | 3.6 | 81.1 |
| Hallucinogens |  |  |  |  |  |  |  |  |  |
| SDS | .60 (.37, .82) | 100.0 | 28.8 | 5.5 | 100.0 | 1.40 | .00 | 5.5 | 28.8 |
| CRAFFT | .69 (.47, .82) | 100.0 | 17.8 | 4.8 | 100.0 | 1.22 | .00 | 4.8 | 17.8 |
| ASSIST-Y | .40 (.13, .67) | 0.0 | 79.5 | 0.0 | 95.1 | .00 | 1.26 | 0.0 | 75.5 |
| Note: Opioids excluded from analysis due to insufficient cases of dependence.  SDS=Severity of Dependence Scale, CRAFFT=CRAFFT screening tool, ASSIST-Y=Alcohol, Smoking and Substance Involvement Screening Test-Youth (15-17 years), CAST=Cannabis Abuse Screening Tool, AUC=Area Under the Curve, Se.=Sensitivity, Sp.=Specificity, PPV=Positive Predictive Value; NPV=Negative Predictive Value, LR+=Likelihood Ratio (positive), LR-=Likelihood Ratio (negative), CUI+=Positive Clinical Utility Index, CUI-=Negative Clinical Utility Index. | | | | | | | | | |
